# Color Stability and Wear Behavior of Polished and Glazed Lithium Aluminium Disilicate Hybrid Abutment Crowns: A 3-Year Clinical Pilot Study

**DOI:** 10.3390/dj14050253

**Published:** 2026-04-27

**Authors:** Jeremias Hey, Carl-Rainer Griesbach, Monika Kasaliyska, Christin Arnold, Ramona Schweyen

**Affiliations:** Department of Prosthodontics and Geriatric Dentistry, Martin Luther University Halle-Wittenberg, Magdeburger Str. 16, 06112 Halle (Saale), Germany

**Keywords:** lithium aluminium disilicate, hybrid abutment crown, dental implants, color stability, surface finishing, wear behavior

## Abstract

**Objectives**: To evaluate the influence of two surface finishing procedures—mechanical polishing and glaze firing—on the color stability and wear behavior of lithium aluminium disilicate (LAD) hybrid abutment crowns over a three-year clinical observation period. **Methods**: Twenty-four patients requiring 34 implant-supported single crowns were included in this prospective clinical study. LAD abutment crowns were fabricated using n!ce ceramic and a CAD/CAM workflow and finished either by mechanical polishing (P, n = 17) or glaze firing (G, n = 17). After insertion as well as after one and three years (P: n = 9, G: n = 9) of clinical use color measurements were performed using spectrophotometry, and color differences (ΔE00) were calculated. Wear was assessed by digital surface comparison of baseline and the two follow-up scans using three-dimensional analysis software. Reference teeth (R) were defined and evaluated comparable to the P and F groups. Biological and technical complications were recorded throughout the observation period. **Results**: Color deviations increased over time in all groups (P, G, R). After three years, G showed lower mean color differences (ΔE00 ≈ 2.77) compared with F (ΔE00 ≈ 5.40), although the difference was not statistically significant. No significant differences in vertical height loss were observed between P and G. One adhesive fracture occurred both in the P and G group, five crowns (P: n = 3, G: n = 2) developed periimplantitis. **Conclusions**: Both polishing and glazing resulted in comparable clinical outcomes regarding color stability, wear behavior, and complication rates. **Clinical significance**: Both finishing protocols might be a reliable option for LAD hybrid abutment crowns.

## 1. Introduction

Over the past decades, patient expectations regarding restorative dental treatments have changed considerably. In addition to functional rehabilitation, patients increasingly demand restorations that provide excellent esthetics while ensuring high biocompatibility and long-term clinical stability. At the same time, efficient treatment protocols and digital workflows have become increasingly important in contemporary prosthetic dentistry, particularly in implant-supported rehabilitations [1,2,3]. Digital technologies, including computer-aided design and computer-aided manufacturing (CAD/CAM), have significantly expanded the possibilities for highly precise and time-efficient fabrication of prosthetic restorations, thereby contributing to improved treatment efficiency and predictability [1,3].

These developments have contributed to a growing preference for metal-free restorative materials. Conventional metal–ceramic restorations present limitations regarding esthetic integration and optical appearance. Consequently, clinical practice and research have increasingly focused on all-ceramic restorative materials [2,4]. Dental ceramics offer excellent biocompatibility, favorable mechanical properties, and optical characteristics that enable a close reproduction of natural tooth structure [5,6].

Among available ceramic materials, lithium silicate ceramics (LSC) have become one of the most widely used restorative materials in prosthetic dentistry [7,8]. LSCs are generally regarded as multicomponent, non-stoichiometric systems and can be classified—based on the predominant microstructural phase(s) [9,10]—into four subgroups: (i) lithium disilicate (LDS; mainly Li_2_Si_2_O_5_), (ii) lithium metasilicate (LM; mainly Li_2_SiO_3_), (iii) zirconia-reinforced lithium silicate (ZLS; biphasic Li_2_SiO_3_/Li_2_Si_2_O_5_), and (iv) lithium aluminium disilicate (LAD; biphasic LiAlSi_2_O_6_/Li_2_Si_2_O_5_). Depending on the specific structure, the LSC subgroups combine high mechanical strength with excellent esthetic properties and demonstrated reliable clinical performance in both tooth-supported and implant-supported restorations so far [2,7]. Typical flexural strength values range from approximately 300 to 420 MPa, with fracture toughness values between 2 and 2.5 MPa·m^1^/^2^. These mechanical characteristics allow LSC restorations to be used for a wide range of indications, including inlays, onlays, veneers, single crowns, and short-span bridges [7,8].

Most commercially available CAD/CAM LSCs (e.g., IPS e.max CAD, VITA Suprinity) are supplied as partially crystallized Li_2_SiO_3_ blocks to enable soft machining, followed by mandatory crystallization and glaze firing (>800 °C) to achieve the Li_2_Si_2_O_5_ phase. This thermal treatment markedly enhances mechanical strength [11].

More recently, fully crystallized LAD blocks (e.g., n!ce) have been introduced to reduce fabrication time [9]. These LADs can be cemented directly after milling without further heating or glaze treatment requirements [12]. Consequently, restorations can be finished by mechanical polishing prior to placement, enabling simplified digital workflows. According to manufacturers and recent investigations, these materials exhibit mechanical and optical properties comparable to conventionally processed lithium disilicate ceramics [8,13]. However, clinical evidence regarding the optimal surface finishing protocol remains limited, and some studies suggested that additional sintering may reduce potential milling-induced surface defects [12].

### 1.1. Influence of Surface Finishing on Ceramic Restorations

Surface characteristics play a crucial role in the long-term clinical performance of ceramic restorations. After milling, ceramic restorations are typically finished either by glazing or mechanical polishing. Both procedures aim to reduce surface roughness and thereby improve mechanical behavior, esthetic appearance, and biological compatibility.

A smooth ceramic surface has been associated with reduced plaque accumulation, improved biocompatibility, and decreased wear of both the restoration and antagonistic enamel [6,14,15,16]. Surface finishing also affects color stability and gloss retention, which are key parameters for the long-term esthetic success of ceramic restorations [17,18].

Laboratory studies frequently report smoother surfaces and higher gloss values after glazing compared with mechanical polishing [17]. However, the durability of the glaze layer remains controversial, particularly following occlusal adjustment or functional loading. Recent investigations suggest that polished surfaces may provide comparable or even superior clinical performance, particularly with regard to maintaining surface smoothness after occlusal adjustments [6]. Experimental studies further indicate that both glazing and polishing can significantly reduce surface roughness in CAD/CAM LSC restorations [19].

### 1.2. Wear Behavior and Antagonistic Enamel Abrasion

Dental wear is considered a multifactorial process influenced not only by intrinsic material properties, such as hardness and fracture toughness, but also by functional loading, parafunctional habits, chemical influences, and surface topography [2,20,21]. Particular attention has been directed toward the potential abrasion of antagonistic enamel caused by ceramic restorations. Several experimental studies indicate that surface finishing procedures may significantly influence the wear behavior of ceramic materials and their interaction with opposing enamel [2,21,22]. Some investigations report reduced antagonistic enamel wear with polished ceramic surfaces compared with glazed surfaces, whereas other studies describe only minimal differences between both finishing approaches [21,22,23]. Recent biomaterials research has also evaluated the influence of surface finishing on wear resistance and fracture load of ceramic crowns. Hoffmann et al. (2024) reported that surface treatments may affect wear behavior, whereas fracture resistance appears to remain largely unaffected [23].

### 1.3. Limitations of Current Evidence

Despite the growing number of experimental investigations, the available evidence regarding the optimal surface finishing protocol for LSC restorations remains inconclusive. Most existing studies are based on in vitro experimental designs employing standardized specimens that do not adequately reproduce the complex biomechanical, chemical, and biological conditions of the oral environment [2,5,6]. Although chewing simulation models provide valuable insights into wear mechanisms and material behavior, they cannot fully replicate the multifactorial conditions present in vivo, including saliva composition, patient-specific occlusal patterns, parafunctional habits, and biological aging processes [15,21]. Consequently, the clinical relevance and transferability of laboratory findings to routine clinical practice remain limited. Clinical studies evaluating the mid-term performance of LSCs, especially LADs, restorations are still relatively scarce. While recent investigations report favorable survival rates of LSC crowns, most studies focus primarily on restoration survival rather than surface finishing protocols or wear behavior. Therefore, reliable clinical evidence comparing polished and glazed LAD restorations remains limited, particularly in implant-supported applications.

### 1.4. Aim of the Study

Although LSCs are widely used in contemporary prosthetic dentistry, there is currently no clear clinical consensus regarding the optimal surface finishing protocol of LAD. In particular, clinical data comparing mechanically polished and glaze-fired LAD restorations with respect to color stability, wear behavior, and clinical complications remain limited.

Therefore, the aim of the present prospective clinical study was to evaluate whether hybrid abutment crowns fabricated from one type of LAD ceramic and finished either by mechanical polishing or glaze firing demonstrate comparable long-term clinical performance regarding color stability, wear behavior, and biological or technical complications. The null hypothesis was that no clinically relevant differences would be observed between the two surface finishing protocols.

## 2. Materials and Methods

### 2.1. Study Design and Patient Recruitment

This clinical pilot study was conducted between 2021 and 2025 at the Department of Dentistry of Martin Luther University Halle-Wittenberg (Halle, Germany). The study protocol was approved by the local ethics committee of the Medical Faculty (approval number: 2019-171, Approval date: 14 February 2020) and performed in accordance with the Declaration of Helsinki. All participants provided written informed consent prior to enrollment.

Patients requiring implant-supported single-crown restorations in the posterior region were screened for eligibility.

Participants were included if the following criteria were fulfilled: stable peri-implant bone and soft-tissue conditions, indication for implant-supported single crowns in the posterior region, adequate oral hygiene and compliance, and age ≥ 18 years.

Patients were excluded if any of the following conditions were present: acute periodontitis or peri-implantitis, xerostomia, diagnosed bruxism or severe parafunctional habits, drug abuse or insufficient compliance, lack of written informed consent, and limited legal capacity or minority status.

### 2.2. Digital Workflow and Prosthetic Treatment

Implant crowns’ fabrication and insertion was performed by two calibrated dentists and an experienced dental technician, all of whom were previously calibrated in the fabrication workflow to minimize inter-operator variability.

Following routine prosthetic pretreatment, an intraoral scan of both jaws and the habitual occlusion was obtained using an intraoral scanner (TRIOS 3; 3Shape, Copenhagen, Denmark) in combination with implant-specific scan bodies (2nd generation scan bodies, C and N series; Medentika, Hügelsheim, Germany). The acquired datasets were exported in STL format and imported into CAD software (3D CARES Visual Software, DWOS platform–based version, Straumann, Freiburg, Germany). Hybrid abutment crowns were digitally designed and milled from LAD ceramic blocks (n!ce glass ceramic HT/C14; Straumann, Freiburg, Germany; Table 1) using a four-axis milling unit (Straumann CARES C series, Straumann, Freiburg, Germany).

After milling, the restorations were separated from the blocks using diamond instruments under continuous water cooling. For clinical try-in, the restorations were temporarily connected to titanium adhesive bases. Marginal fit, proximal contacts, and static and dynamic occlusion were adjusted intraorally.

### 2.3. Surface Finishing Protocols

After clinical adjustment, the n!ce-ceramic hybrid crowns were randomly distributed between two surface-finishing protocols: polishing and glazing. When patients required two or more hybrid implant crowns, both polishing and glazing protocols were applied.

#### 2.3.1. Polishing

Polishing was performed using the OptraFine diamond polishing system (Ivoclar Vivadent AG, Schaan, Liechtenstein). After occlusal and proximal adjustments, the restoration surface was cleaned to ensure a uniform baseline. Pre-polishing was carried out with fine-grit stage F instruments (Flame, Cup, Disc) at 7000–12,000 rpm to reduce roughness and remove grinding marks, with shape-specific application depending on surface geometry. Subsequently, high-gloss polishing was performed using stage P instruments (5000–10,000 rpm) following the same systematic sequence. Final polishing was achieved using the OptraFine HP nylon brush (Ivoclar Vivadent AG, Schaan, Liechtenstein) with polishing paste at low speed (3000–5000 rpm) under minimal pressure. The crown was then thoroughly cleaned, resulting in a homogeneous, defect-free, high-gloss surface.

#### 2.3.2. Glaze Firing

In the second group, restorations were finally glaze fired using the ceramic glaze system recommended for n!ce ceramic according to the manufacturer (n!ce Glaze & 3D AddOn Kit; Straumann, Freiburg, Germany) in a ceramic furnace (Vario Press 200; Zubler, Ulm, Germany) according to the manufacturer’s recommendations.

After surface treatment, both polished as well as glaze-fired ceramic superstructures were adhesively bonded to the titanium bases using a dual-curing resin cement (Multilink Hybrid Abutment; Ivoclar Vivadent). The restorations were subsequently inserted and screw-retained according to the manufacturer’s recommendations.

### 2.4. Color Determination

Color measurements were performed immediately after hybrid implant crowns insertion and at follow-up visits after 12 and 36 months. Color was determined using a spectrophotometer (Easyshade V; VITA Zahnfabrik, Bad Säckingen, Germany). Prior to each measurement, the spectrophotometer was calibrated using its internal ceramic reference standard. Calibration was performed by placing the probe tip into the designated calibration port and initiating the automatic calibration cycle. All measurements were carried out under standardized ambient lighting conditions. The measurement tip was positioned perpendicular (90°) to the tooth surface to minimize angular discrepancies. The tooth surfaces were cleaned and gently air-dried prior to measurement to eliminate contaminants and surface moisture. To standardize the measurement location, individualized thermoformed templates with a defined measurement window were fabricated for each patient and measurements were performed by a two calibrated operators. For each hybrid implant crown and reference tooth, three consecutive measurements were recorded at the middle third of the labial surface, and the mean values were calculated to reduce variability. Color parameters were obtained in the CIE Lab* color space system.

Color differences were calculated using the CIEDE2000 formula (ΔE00) between baseline and follow-up measurements after 12 and 36 months of clinical use (Section 2.7).

### 2.5. Wear Measurement

To evaluate the wear behavior after different surface finishing protocols, the vertical material loss of the restorations and the corresponding antagonist teeth was assessed over time. For this purpose, double-mix impressions of the test arch and the opposing arch were taken using individual impression trays and polyether impression material (Impregum/Permadyne; 3M Solventum, Kamen, Germany) directly after implant crowns’ insertion and after 12 and 36 months of clinical use by two calibrated dentists. The impressions were carefully rinsed and immediately poured with type IV dental stone (Original Rocky Mountain IV; Dental, Augsburg, Germany) according to the manufacturer’s instructions to produce precision gypsum models. The resulting models were subsequently digitized using an intraoral scanner (Trios 3, 3Shape, Copenhagen, Denmark) by one single investigator. Accordingly, datasets were collected at baseline immediately after crown insertion, as well as during follow-up examinations at 12 and 36 months of clinical service.

The resulting STL datasets were used for quantitative wear analysis performed by one single operator. To enable comparison between baseline and the two follow-up models, the digitized datasets were virtually superimposed using inspection software (GOM Inspect Pro 2021; Carl Zeiss GOM Metrology, Braunschweig, Germany). Because morphological changes of the jaws and potential dental interventions may affect the geometry of the entire dentition over extended observation periods, a localized alignment protocol was applied instead of a full-arch matching approach. Preliminary analyses demonstrated that the commonly used root mean square (RMS) deviation method was not suitable for accurate alignment of the datasets, as physiological skeletal remodeling and age-related bone resorption may alter the overall jaw geometry over time. Such changes may influence both the maxilla and mandible and can result in multidimensional morphological variations that could compromise full-arch matching procedures. Therefore, the matching process was performed in several sequential steps. First, a baseline STL dataset was imported into the software and defined as the reference model. Follow-up scans obtained at the respective recall appointments were subsequently imported into the same project environment. An initial global pre-alignment was performed to establish a preliminary spatial correspondence between the baseline and follow-up datasets. In cases of pronounced morphological variation, the digital models were additionally segmented into smaller anatomical regions (e.g., jaw quadrants) to improve alignment accuracy. Subsequently, a local best-fit registration was performed using predefined reference areas. Vestibular and oral tooth surfaces were primarily selected as reference regions because these areas were assumed to undergo minimal morphological alterations during the observation period. Based on these reference areas, the follow-up scans were precisely superimposed onto the baseline dataset. Finally, the alignment between the baseline model and the follow-up meshes was visually inspected and corrected when necessary to ensure accurate superimposition prior to quantitative wear analysis.

**Figure 1:**
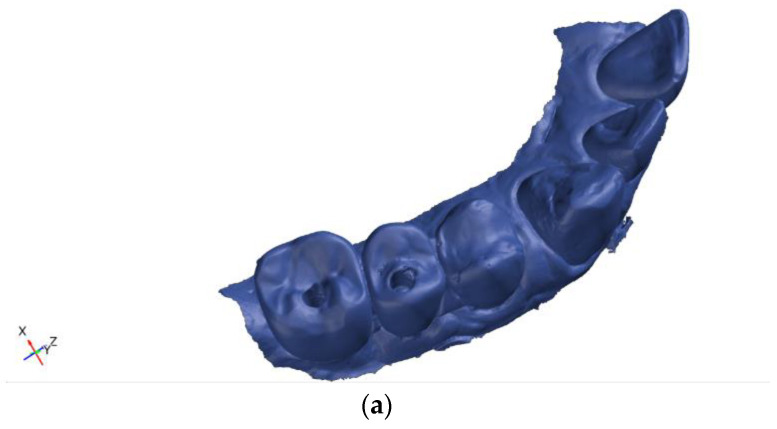

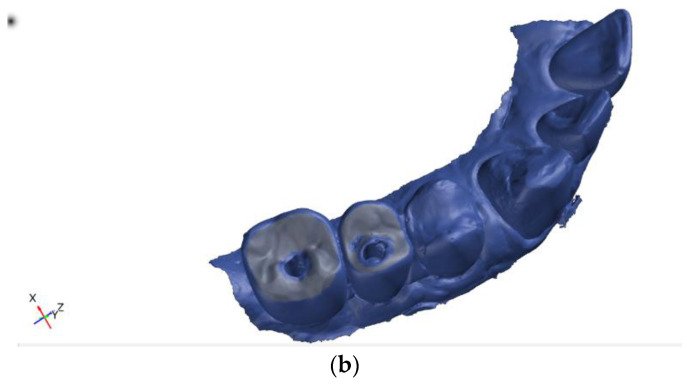
GOM Inspect Pro 2021-based measurement workflow. (**a**) Baseline model represented as a CAD object; (**b**) definition of the occlusal surface used as the measurement key for wear analysis.

The occlusal surface to be analyzed was selectively defined on the baseline CAD model as a measurement area (Figure 1b). Regions expected to undergo significant structural changes, such as screw access channels and their margins, were excluded from the analysis to avoid potential measurement artifacts. The definition of the measurement area was performed individually for each tooth and repeated accordingly for all evaluated restorations.

**Figure 2:**
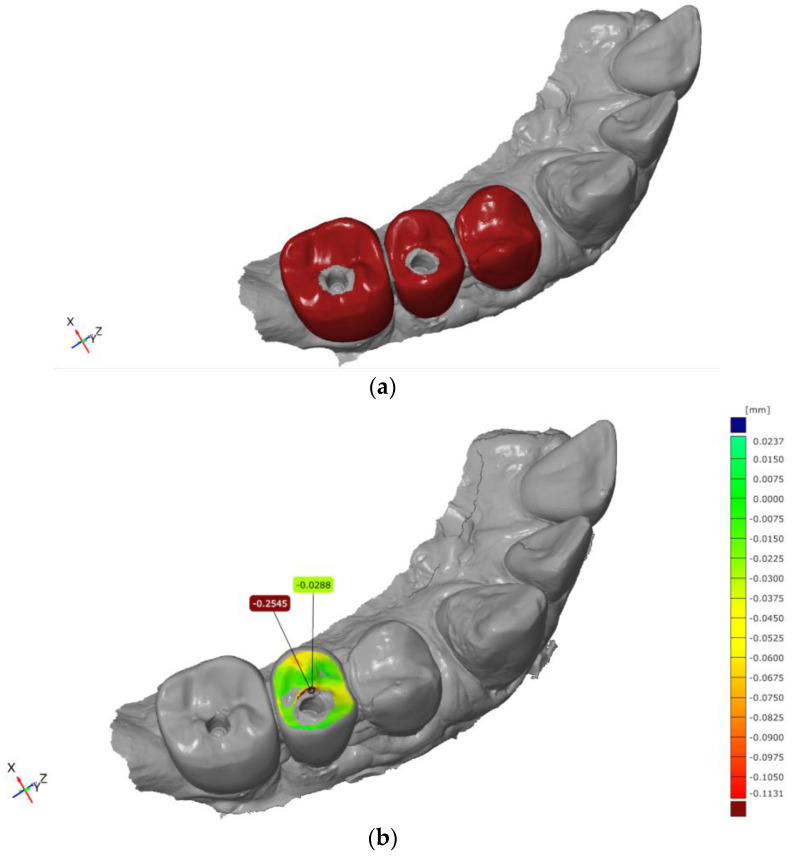
Wear analysis using GOM Inspect Pro 2021 software after 1 year: (**a**) local best-fit surface registration; (**b**) abrasion analysis displayed as a color-coded deviation map of the tooth surface.

**Figure 3:**
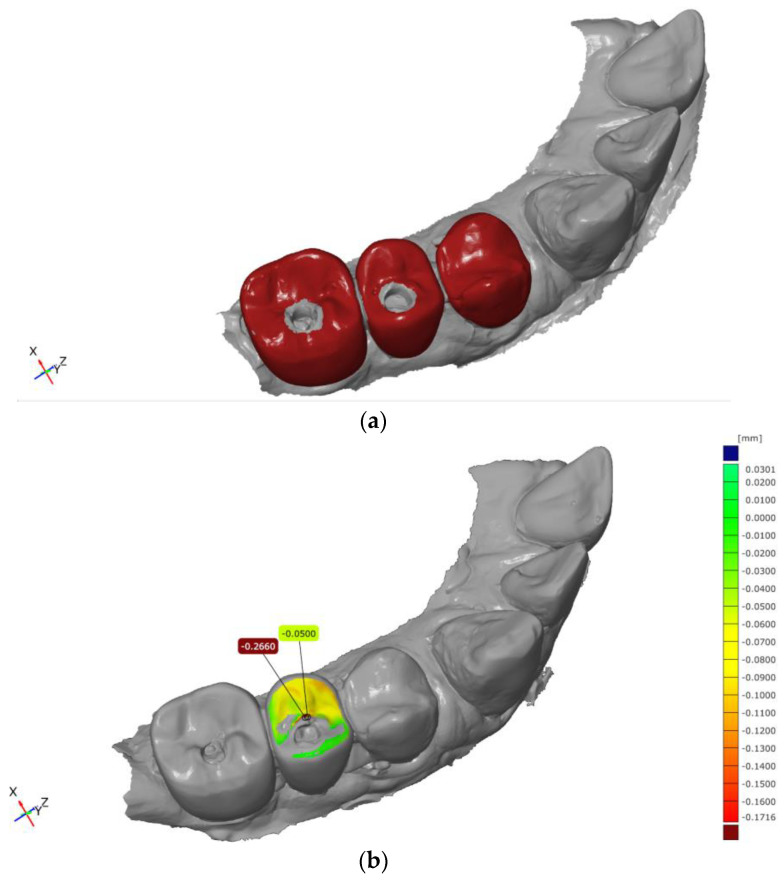
Wear analysis using GOM Inspect Pro 2021software after 3 years: (**a**) local best-fit surface registration; (**b**) abrasion analysis displayed as a color-coded deviation map of the tooth surface.

For quantitative assessment, an area-based comparison was performed between the baseline CAD dataset and the corresponding follow-up scan. This procedure generated a color-coded deviation map visualizing dimensional differences between the datasets (Figure 2 and Figure 3). The deviation scale was adjusted to allow clear visualization of material loss, with areas of increased abrasion displayed in warm colors (e.g., yellow to red), enabling a differentiated representation of the observed surface changes.

In addition to the qualitative visualization, quantitative parameters were calculated for each defined measurement area. These included maximum deviation values, arithmetic mean deviation, and the range of the measured differences.

To evaluate the reliability of the measurement procedure (intra-rater reliability), the scanning and analysis workflow was additionally performed three times on an independent plaster model outside the clinical study. The analysis was conducted for a representative tooth, and the mean deviation values of the repeated scans were calculated to assess potential measurement variability related to the scanning and analysis process.

### 2.6. Biological and Technical Complications

At each follow-up visit, the hybrid crowns were clinically examined by visual inspection, and peri-implant probing depths were recorded using a WHO periodontal probe. The occurrence of biological complications, including gingivitis and peri-implantitis, as well as technical complications, such as cracks, chipping, crown fracture, loss of retention, screw loosening, and screw fracture, was documented. Cracks were defined as superficial defects of the ceramic veneer that became visible under varying angles of incident light without associated material loss. Chipping was defined as localized loss of ceramic material accompanied by visible structural defects and discoloration in the affected area. All biological and technical complications occurring during the entire observation period were systematically recorded and documented.

### 2.7. Statistical Analysis

Descriptive statistics were calculated for all variables. Prior to inferential analysis, the data were tested for normal distribution using the Kolmogorov–Smirnov test and for homogeneity of variances using Levene’s test. As the assumptions of normal distribution and homogeneity of variances were not met, non-parametric statistical methods were applied. Group comparisons were performed using the Mann–Whitney U test and the Kruskal–Wallis test, as appropriate. All statistical analyses were conducted using statistical software (IBM SPSS Statistics, version 31.0; IBM, Ehningen, Germany). Results were considered statistically significant at *p* ≤ 0.05.

*Primary outcome: color differences*

In the CIEDE2000 (ΔE00) color difference formula, perceptual non-uniformities of the CIELab color space are corrected using weighting functions for lightness (SL), chroma (SC), and hue (SH). In addition, parametric correction factors (KL, KC, and KH) are applied to account for environmental and viewing conditions. Differences in lightness, chroma, and hue between two measurements are expressed as ΔL, ΔC, and ΔH, respectively.

In this study, the ΔE00 values between the initial measurement after insertion and the follow-up measurements were calculated using

$$\Delta E_{0_{0}}=\sqrt{{\left(\frac{\Delta L^{\prime }}{k_{L}S_{L}}\right)}^{2}+{\left(\frac{\Delta C^{\prime }}{k_{C}S_{C}}\right)}^{2}+{\left(\frac{\Delta H^{\prime }}{k_{H}S_{H}}\right)}^{2}+R_{T}\left(\frac{\Delta C^{\prime }}{k_{C}S_{C}}\right)\left(\frac{\Delta H^{\prime }}{k_{H}S_{H}}\right)}$$

A maximum of three ΔE00 values were calculated for each hybrid crown based on the respective follow-up measurements. Differences between the three groups (reference teeth, polished crowns, and glazed crowns) at each measurement time point, as well as differences between the measurement time points within each group, were analyzed using the Kruskal–Wallis test.

*Secondary endpoints: wear and complications*

Secondary outcomes included mean vertical wear, expressed as the average vertical height loss, as well as the occurrence of technical and biological complications.

Differences in wear between the study groups were analyzed analogously to the color evaluation using non-parametric statistical tests (Mann–Whitney U test and Kruskal–Wallis test), as appropriate. Technical and biological complications were analyzed descriptively.

## 3. Results

### 3.1. Study’s Cohort

**Figure 4:**
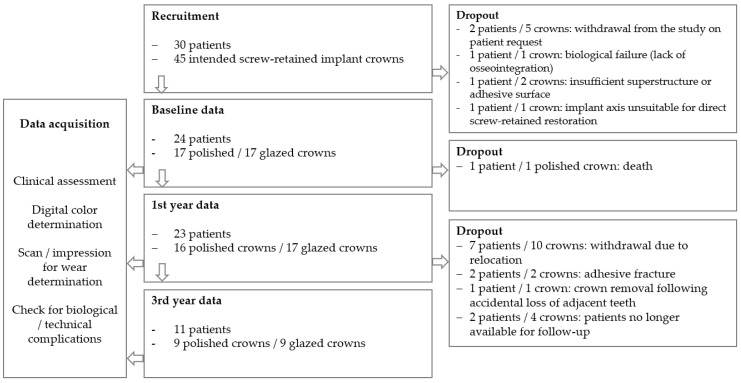
Flowchart showing the study process with reasons for premature withdrawal of patients and crowns.

Initially, 30 patients requiring a total of 45 hybrid crowns were enrolled in the study. Baseline data were obtained from 24 patients with 34 hybrid implant crowns made of n!ce ceramic (17 polished and 17 glazed). A total of 18 crowns (9 polished and 9 glazed) were followed over a three-year observation period. The reasons for premature withdrawal from the study are presented in Figure 4.

### 3.2. Discoloration

Descriptive analyses of the ∆E00-values between the baseline, 1st year and 3rd year digital color measurements are presented in Table 2.

Comparison of color deviations between baseline measurements and follow-up assessments revealed no statistically significant differences among the three groups after 1 year (*p* = 0.208). After 3 years, lower color deviations were observed for the glazed crowns (ΔE00 = 2.77 ± 3.2) compared with the polished crowns (ΔE00 = 5.40 ± 2.86); however, this difference did not reach statistical significance (*p* = 0.074).

**Figure 5:**
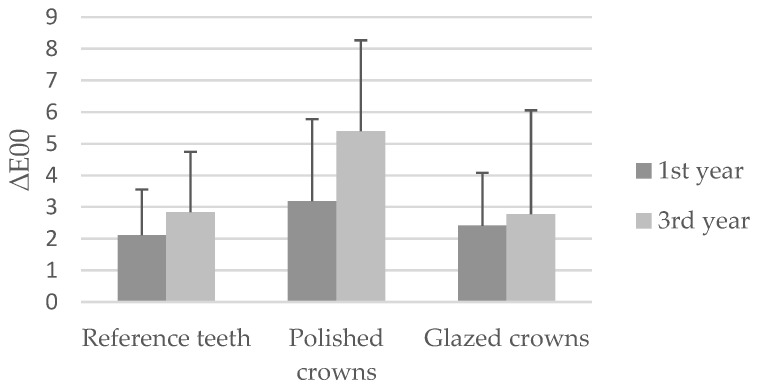
Color deviations (ΔE00) between baseline and follow-up measurements for the different study groups.

The distribution of the calculated color deviations for the respective groups over the observation periods is presented in Figure 5.

### 3.3. Wear and Vertical Height Loss

Descriptive analyses of the average and maximum vertical height loss after the 1st year and 3rd year of clinical use are presented in Table 3.

**Figure 6:**
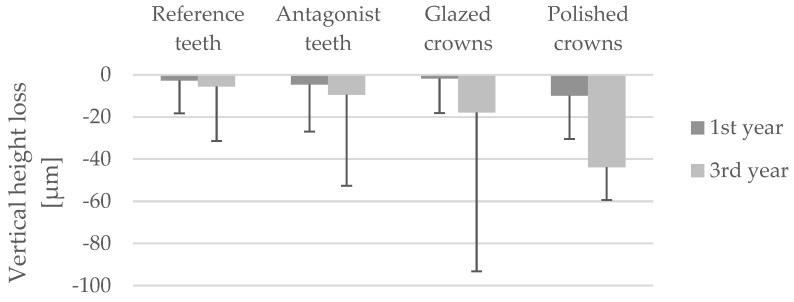
Vertical height loss in the different study groups during the observation period (µm, mean ± SD).

The vertical height loss found in the three groups provided wide variances (Figure 6).

No statistically significant differences in mean vertical height loss were observed either between the study groups at the respective measurement time points (Kruskal–Wallis test, *p* > 0.05) or between the different observation times within each group (Wilcoxon signed-rank test, *p* > 0.05).

Repeated measurements performed six months apart to assess intra-rater reliability on a single model resulted in measurement deviations of 3 ± 11 µm for the same examiner.

### 3.4. Biological and Technical Complications

Overall, few biological and technical complications occurred during the observation period (Table 4). No difference was found between the two groups.

## 4. Discussion

The present clinical study evaluated the influence of two surface finishing procedures—mechanical polishing and glaze firing—on the color stability and wear behavior of LAD hybrid abutment crowns over a three-year observation period.

### 4.1. Discoloration

Color changes were observed in all investigated groups, including natural reference teeth. After three years, the glazed crowns exhibited discoloration comparable to that of natural teeth, whereas the polished crowns showed more pronounced discoloration (ΔE00 = 5.40 ± 2.86), although this difference did not reach statistical significance (*p* = 0.074). Consequently, the first part of the null hypothesis—stating that the surface finishing procedure does not significantly influence color stability—can be accepted.

Color differences were calculated using the CIEDE2000 (ΔE00) formula, which has been demonstrated to correlate more closely with human visual perception than the traditional CIELAB color difference formula and is therefore recommended for the evaluation of dental materials [24]. Earlier studies reported perceptibility and acceptability thresholds of ΔE00 = 1.28 and ΔE00 = 2.24 [25], whereas later investigations suggested more stringent thresholds of approximately ΔE00 = 0.8 and ΔE00 = 1.8 for perceptibility and clinical acceptability, respectively [24]. In this study, all investigated groups exceeded the acceptability threshold after one year. However, comparable color changes were observed between glazed crowns and the natural reference teeth. This finding indicates that discoloration cannot be attributed solely to the restorative materials but is likely influenced by intraoral environmental factors. Dietary habits, smoking, oral hygiene, and biofilm accumulation have all been reported to contribute significantly to the long-term discoloration of restorative materials [26,27]. Nevertheless, a clinically perceptible difference in discoloration between the polished and glazed crowns was observed after three years, exceeding the thresholds reported in the literature as being clinically relevant.

Material composition is a key determinant of color stability. LSCs in general as well as LADs in particular are supposed to exhibit superior optical stability compared with resin-based restorative materials due to their dense crystalline microstructure and reduced water sorption [6,28,29,30,31]. Recent investigations evaluating CAD/CAM ceramics confirmed that LSC materials demonstrate favorable optical properties and relatively stable color behavior after artificial aging procedures, especially in comparison to resin nanoceramics [30,32,33].

However, besides material composition, surface finishing procedures may further affect the susceptibility of ceramic restorations to discoloration. Glazing produces a thin glassy surface layer that reduces surface roughness and limits the retention of staining agents. In contrast, polishing procedures may leave microscopic surface irregularities that can facilitate staining over time. Other studies reported slightly higher discoloration values for polished surfaces compared with glazed surfaces after artificial aging procedures [30,34]. The tendency observed in the present clinical study—higher ΔE00 values for polished crowns—therefore appears to be consistent with previously published experimental findings. However, it has to be taken into account that, beside the ceramic type used, also the ageing process of resin cements was found to influence the optical properties of cemented ceramic restorations [35]. As for both types of hybrid crowns the same resin cement was used, its potential influence on discoloration might be considered as comparable. Other experimental studies investigating CAD/CAM ceramics also demonstrated that material composition, thickness, and exposure to staining media significantly influence the color stability and translucency of LSCs [31,32,33,34,36]. As no evaluation about hybrid crowns’ thickness was performed in the present study, no conclusion can be drawn concerning its potential influence on color stability.

Overall, the findings about discoloration support the assumption that both intrinsic material characteristics and extrinsic environmental factors contribute to mid-term color changes of LAD hybrid abutment crowns. However, given the relatively small study cohort, these findings should be interpreted as preliminary trends and warrant critical re-evaluation in future prospective studies with larger patient populations.

### 4.2. Biological and Technical Complications

During the observation period, only a limited number of biological and technical complications were recorded, and no relevant differences were detected between polished and glazed crowns. Biological complications such as gingivitis or peri-implantitis occurred rarely and were evenly distributed between the study groups. Technical complications were also infrequent and mainly consisted of isolated adhesive fractures. This observation is in line with results of the literature that found no difference in flexural strength between glazed and polished lithium disilicate [37]. In the present study, one fracture occurred during the extraction of an adjacent tooth and therefore cannot be attributed to the restorative material itself. In another case, no fabrication error or adhesive failure could be identified. Potential “biological” causes such as excessive occlusal loading, premature contacts, or parafunctional habits cannot be completely excluded. Because only a brief functional screening was performed prior to patient inclusion, mechanical overload may have contributed to the observed failure.

### 4.3. Wear Behavior

The mean vertical height loss observed during the study period ranged from −1.7 ± 16.3 µm and −43.8 ± 156.3 µm. No statistically significant differences were detected either between the investigated materials or between the different examination time points within the respective groups. Accordingly, the second part of the null hypothesis—stating that the surface finishing protocol does not significantly influence wear behavior—can be accepted.

To date, few studies have specifically compared the clinical wear behavior of polished or glazed LSC, especially LAD, hybrid abutment crowns. The wear values reported in the literature vary considerably depending on the experimental design and measurement methodology. In vitro investigations reported vertical height losses of 132.2 ± 19.9 µm [29] and 264.3 ± 56.1 µm [38]. Clinical studies, in contrast, reported vertical height losses of approximately 78 µm after four years [39] and 148 µm after one year [40]. Physiological enamel abrasion was found to be between 15 µm and 29 µm in the posterior region within four years [41]. The wear values observed in the present study are therefore generally consistent with the range reported in the literature, particularly when the inherent measurement uncertainties associated with the applied methodology are considered. Wulfman et al. (2018) demonstrated that most wear studies rely on replicas derived from polyvinylsiloxane impressions cast in type IV dental stone [42]. A comparable workflow was used in the present study. Previous investigations reported a linear accuracy of approximately 9 µm for this method, depending on impression technique and tray selection [43]. Nevertheless, detailed information regarding parameters influencing scanning precision—such as laser spot size, spatial resolution, scanning step size, and sample angulation—remains limited. According to Wulfman et al. (2018), the lateral resolution (x/y) in most studies ranges between 20 and 25 µm, with similar scanning step sizes, whereas the vertical resolution (z-axis) of laser scanners typically ranges from 5 to 9 µm [42]. Based on these parameters, the overall accuracy of digital wear assessment workflows has been estimated to lie between approximately 15 and 20 µm [42,44,45]. Moreover, intra-rater reliability has to be taken into account, that was found to be 3 ± 11 µm in the present study.

Consequently, a proportion of the variance observed in the measured wear values may be attributable to methodological limitations rather than to true differences in material wear. Moreover, some patients received two implant-supported hybrid crowns, one finished by polishing and the other by glazing, introducing the potential for differential wear and discoloration influenced by individual parafunctional habits (e.g., bruxism). This may have affected the statistical outcomes, as all crowns were treated as independent observations. Moreover, no surface roughness measurements were performed in the present study. As discussed above, occlusal surface roughness is assumed to influence subsequent wear, particularly of the antagonist dentition. Future studies should therefore include surface roughness analyses to critically evaluate the integrity of both polished and glazed surfaces as well as the corresponding antagonists after several years of clinical function. Furthermore, the high dropout rate during the follow-up period, associated with the COVID-19 pandemic, resulted in a limited number of restorations being available for evaluation at the three-year follow-up. Accordingly, the generalizability of the findings is limited and should be interpreted with caution, taking into account the pilot nature of the present study.

Nevertheless, clinical data comparing polished and glazed LAD hybrid abutment crowns remain scarce. Thus, the present pilot study provides first valuable clinical evidence regarding the performance of these restorations. However, as noted above, further prospective clinical studies with larger patient cohorts and extended follow-up periods are required to comprehensively evaluate the long-term optical stability and wear behavior of LAD in implant-supported prosthetic reconstructions.

## 5. Conclusions

Within the limitations of this clinical pilot study, both surface finishing procedures—mechanical polishing and glaze firing—applied to LAD hybrid abutment crowns demonstrated comparable outcomes in terms of color stability, wear behavior, and the incidence of biological and technical complications over a three-year observation period. Polished crowns tended to exhibit greater discoloration compared to glazed crowns. Thus, both finishing approaches appear to be suitable for LAD restorations. However, further clinical investigations with larger patient cohorts and extended follow-up periods are necessary to confirm these findings and to more comprehensively assess the long-term optical and mechanical performance of these materials.

## Figures and Tables

**Table 1:** Composition of n!ce ALD ceramic according to the manufacturer’s information.

SiO_2_	Li_2_O	Al_2_O_3_	Na_2_O	K_2_O	P_2_O_5_	ZrO_2_	CaO	Oxide Colorants
64–70%	10.5–12.5%	10.5–11.5%	1–3%	0–3%	3–8%	0–0.5%	1–2%	0–9%

**Table 2:** Descriptive analyses of the ∆E00-values between the baseline, 1st year and 3rd year digital color measurements.

Evaluated Object	1st Year					3rd Year				
	n	Mean ± SD	Mdn	Max	Min	n	Mean ± SD	Mdn	Max	Min
Reference teeth	23	2.10 ± 1.47	1.82	6.32	0.40	11	2.84 ± 1.90	2.25	6.53	0.70
Polished crowns	17	3.18 ± 2.59	2.48	10.01	0.26	9	5.40 ± 2.86	4.76	10.16	1.77
Glazed crowns	18	2.41 ± 1.67	1.90	6.65	0.70	9	2.77 ± 3.28	0.87	10.68	0.62

**Table 3:** Vertical wear of restorations, reference teeth, and antagonists after 1 and 3 years expressed as mean and maximum vertical height loss (µm).

Evaluated Object	1st Year			3rd Year		
	n	Mean Vertical Height Loss (µm, Mean ± SD)	Maximum Vertical Height Loss (µm, Mean ± SD)	n	Mean Vertical Height Loss (µm, Mean ± SD)	Maximum Vertical Height Loss (µm, Mean ± SD)
Reference teeth	21	−2.8 ± 15.5 µm	−191.0 ± 152.1 µm	11	−5.5 ± 25.9 µm	−248.7 ± 164.1 µm
Polished crowns	16	−9.8 ± 20.6 µm	−171.4 ± 195.4 µm	9	−43.8 ± 156.3 µm	−270.9 ± 263.9 µm
Glazed crowns	17	−1.7 ± 16.3 µm	−166.8 ± 265.5 µm	9	−17.9 ± 75.3 µm	−143.3 ± 70.48 µm
Antagonist teeth	32	−4.6 ± 22.3 µm	−183.8 ± 186.7 µm	18	−9.5 ± 43.2 µm	−239.0 ± 365.8 µm

**Table 4:** Technical and/or biological failures during the study period.

Variable	Polished Crowns	Glazed Crowns
Biological complications (gingivitis, peri-implantitis)	2 patients/3 crowns	2 patients/2 crowns
Crown infractions	–	–
Adhesive crown fracture	1 (spontaneous)	1 (during lever extraction of an adjacent tooth)
Cohesive crown fracture	–	–

## Data Availability

The datasets generated and analyzed during the current study are available from the corresponding author on reasonable request.

## Abbreviations

The following abbreviations are used in this manuscript:

ΔE00	Delta E, color difference
a	Green–red axis in the CIE Lab* color space
b	Yellow–blue axis in the CIE Lab* color space
ΔL′	Corrected lightness difference
ΔC′	Corrected chroma difference
ΔH′	Corrected hue difference
C	Chroma
CAD	Computer-Aided Design
CAM	Computer-Aided Manufacturing
CIE	Commission Internationale de l’Éclairage
CIEDE2000	Color difference formula
e.g.	for example
ISO	International Organization for Standardization
k_L_, k_C_, k_H_	parametric weighting factors for lightness, chroma, and hue
L	Lightness
MPa	Megapascal
m	Metre
µm	Micrometre
n	Number
*p*	Probability value
RMS	Root mean square
SD	Standard deviation
S_L_, S_C_, S_H_	Weighting functions for lightness, chroma, and hue
STL	Standard Tessellation Language
R_T_	Rotation term accounting for the interaction between chroma and hue
WHO	World Health Organization
